# Hepatosplenic Tuberculosis Mimicking Metastatic Malignancy and Immune Reconstitution Inflammatory Syndrome (IRIS) in an HIV-Positive Patient

**DOI:** 10.7759/cureus.100982

**Published:** 2026-01-07

**Authors:** João Pedro Maldonado, Vânia Almeida, Rubén Carvalho, Maria Augusta Cipriano

**Affiliations:** 1 Pathology, Unidade Local de Saúde de Coimbra, Coimbra, PRT; 2 Infectious Diseases, Unidade Local de Saúde de Coimbra, Coimbra, PRT

**Keywords:** extrapulmonary tuberculosis (eptb), hepatic tuberculosis (hepatic tb), histopathology, hiv-positive, percutaneous liver biopsy

## Abstract

A man in his late 30s, recently diagnosed with HIV-1, presented with persistent fever, weight loss, and fatigue, despite prior treatment for immune reconstitution inflammatory syndrome (IRIS). Imaging studies revealed splenomegaly, hepatic and splenic micronodular lesions, and pulmonary micronodules. A liver biopsy, pivotal in establishing the diagnosis, demonstrated multiple well-formed epithelioid granulomas with focal central necrosis, indicative of an infectious etiology. Polymerase chain reaction (PCR) analysis of the biopsy fragment confirmed the presence of *Mycobacterium tuberculosis*. The integration of histopathological findings and PCR analysis was instrumental in diagnosing extrapulmonary tuberculosis (TB) involving the liver and spleen. The patient was started on anti-TB therapy in conjunction with continued antiretroviral treatment, resulting in significant clinical improvement, with resolution of fever and weight gain. This case highlights the critical role of pathology in diagnosing extrapulmonary TB in HIV-positive patients presenting with systemic symptoms such as fever and weight loss.

## Introduction

Extrapulmonary tuberculosis (TB), while less common than pulmonary TB, is a significant manifestation, particularly in immunocompromised individuals, such as those with HIV [1-3]. In Portugal, the incidence of TB was reported at 16 cases per 100,000 people in 2023 according to the World Health Organization (WHO) [4], highlighting the ongoing public health burden of the disease. Its presentation often mimics conditions like malignancies or fungal infections [1,5]. Nonspecific symptoms, including fever and weight loss, as well as systemic involvement, can obscure the diagnosis, particularly when routine diagnostic tests yield negative results. This case highlights the crucial role of an early liver biopsy in the diagnostic workup, enabling an accurate diagnosis and a timely initiation of appropriate therapy.

## Case presentation

A man in his 30s, recently diagnosed with HIV (CD4+ count: 172 cells/μL), presented with a four-day history of fever reaching 39°C, muscle aches, fatigue, weight loss, and night sweats. Two months earlier, he had been hospitalized for a febrile illness thought to be associated with immune reconstitution inflammatory syndrome (IRIS) following the initiation of antiretroviral therapy (bictegravir 50 mg + emtricitabine 200 mg + tenofovir alafenamide 25 mg, once daily). His fever briefly resolved with corticosteroids (prednisolone) but recurred, accompanied by progressive anorexia and weight loss.

His medical history included type 2 diabetes mellitus, diagnosed several years prior, and gastroesophageal reflux disease. He lived with his spouse and reported no significant occupational or travel exposure. He denied any prior history of TB or known exposures. On physical examination, he exhibited considerable weight loss but had no lymphadenopathy, hepatosplenomegaly, ascites, or neurological deficits.

**Figure 1:**
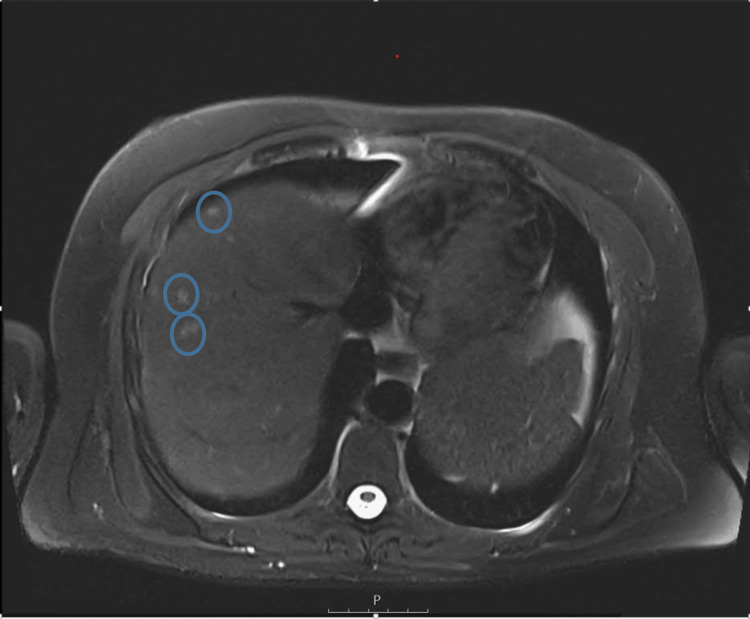
Axial T2-weighted MRI of the upper abdomen Axial T2-weighted MRI of the upper abdomen showing the liver with multiple hyperintense micronodular lesions scattered across both lobes

Initial laboratory results showed elevated inflammatory markers and liver enzymes (Table 1). Imaging studies, including abdominal magnetic resonance imaging (MRI), revealed multiple micronodules in the liver, spleen, and lungs, suggesting a disseminated disease process (Figure 1). Bronchoalveolar lavage and serological tests ruled out pathogens such as *Pneumocystis jirovecii *and Cytomegalovirus.

**Table 1:** Summary of this patient’s blood biochemistry LDH: lactate dehydrogenase; AST: aspartate aminotransferase; SGOT: serum glutamic-oxaloacetic transaminase; ALT: alanine aminotransferase; GPT: glutamic-pyruvic transaminase; GGT: gamma-glutamyl transferase

Parameter	Result	Normal range
Glucose	224 mg/dL	60-109 mg/dL
Urea nitrogen (Azoto uréico)	14.8 mg/dL	7.9-20.9 mg/dL
Creatinine	1.10 mg/dL	0.72-1.18 mg/dL
Sodium	135 mmol/L	136-146 mmol/L
Potassium	4.5 mmol/L	3.5-5.1 mmol/L
Total proteins	7.3 g/dL	6.6-8.3 g/dL
Calcium	10.2 mg/dL	8.8-10.6 mg/dL
Osmolality	278 mOsm/kg	260-302 mOsm/kg
LDH	250 U/L	<248 U/L
AST (GOT)	37 U/L	<35 U/L
ALT (GPT)	51 U/L	<45 U/L
Alkaline phosphatase	302 U/L	30-120 U/L
GGT	669 U/L	<55 U/L
Total bilirubin	1.0 mg/dL	0.2-1.2 mg/dL
Creatine kinase	17.1 U/L	<171 U/L
C-reactive protein	11.92 mg/dL	<0.50 mg/dL

**Figure 2:**
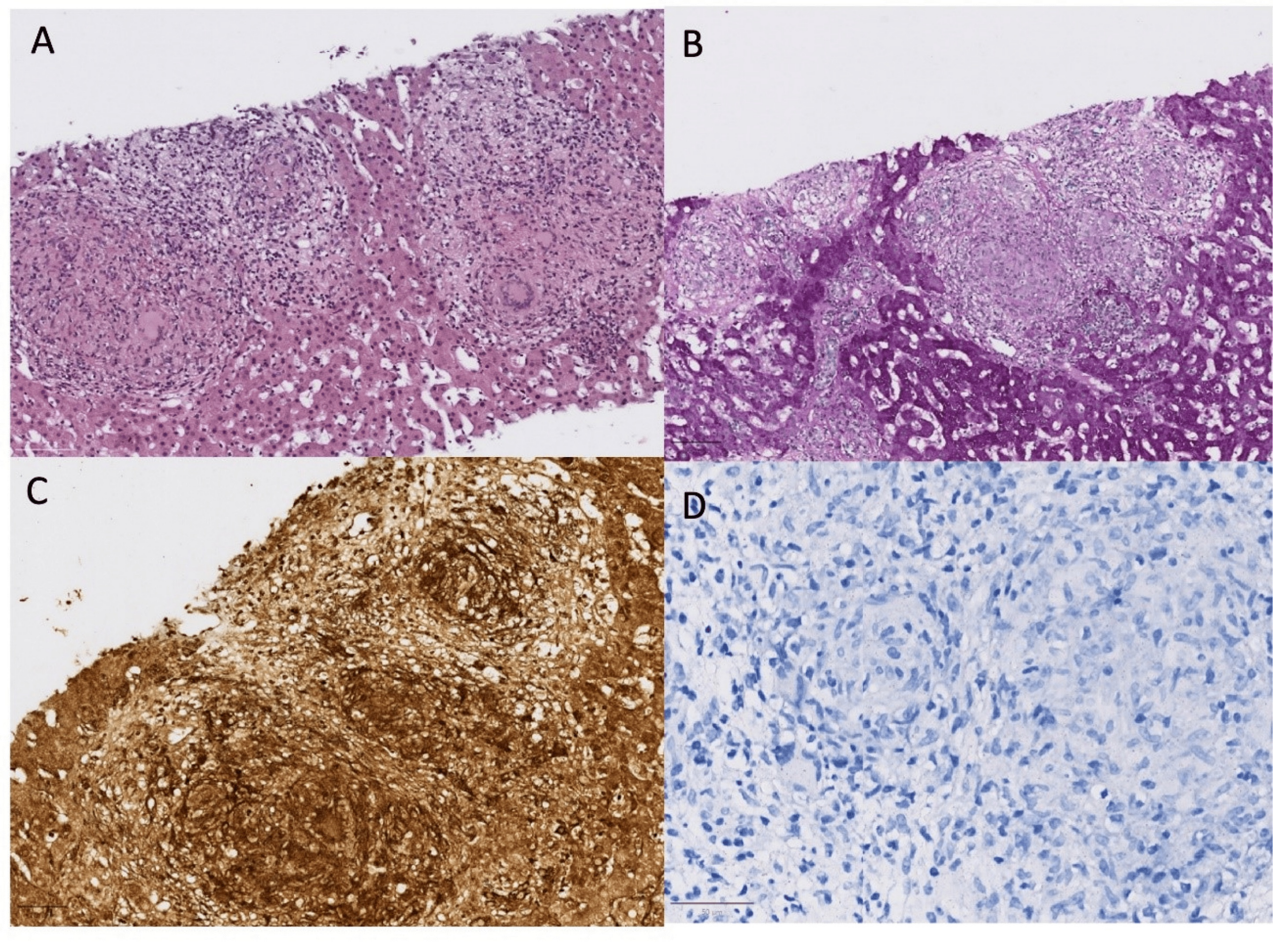
Liver biopsy AFB: acid-fast bacillus; PAS: Periodic Acid-Schiff Liver biopsy showing granulomatous inflammation with multiple well-formed epithelioid granulomas, some displaying central "clean" necrosis (Figure A). There was no evidence of any organism in the AFB stain (Figure B), the Warthin-Starry stain (Figure C), and the PAS stain (Figure D)

A targeted liver biopsy of the micronodules revealed a normal liver architecture with marked lobular disarray due to multiple well-formed sarcoid-type granulomas, some displaying central "clean" necrosis. The granulomas consisted of epithelioid cells, multinucleated Langhans-type giant cells, and peripheral lymphocytes. Despite negative histochemical staining results, including Gram, Periodic Acid-Schiff (PAS), Grocott, Warthin-Starry, and Ziehl-Neelsen techniques, morphological features strongly suggested an infectious etiology (Figure 2). Nucleic acid extraction and polymerase chain reaction (PCR) analysis for *Mycobacterium tuberculosis* confirmed the presence of the bacterium in the biopsy sample

The patient was initiated on a standard anti-TB regimen (isoniazid, rifampin, pyrazinamide, and ethambutol), which led to significant clinical improvement, including the resolution of fever. He remains under close follow-up with ongoing antiretroviral and anti-TB therapy.

## Discussion

Hepatic and splenic TB is rare, accounting for less than 1% of all TB cases [1,3]. It is particularly uncommon for these forms of TB to serve as the initial presentation of the disease, especially in HIV-positive patients [2,6]. Immunosuppression caused by HIV increases susceptibility to latent TB reactivation and dissemination to atypical sites like the liver and spleen [1,5]. In this case, the presence of multiple micronodules across the liver, spleen, and lungs, combined with microbiological confirmation of *Mycobacterium tuberculosis*, led to the assumption of disseminated TB. This case underscores the importance of considering extrapulmonary TB in the differential diagnosis of HIV-positive patients, particularly when symptoms like fever and weight loss mimic other systemic infections or malignancies.

Imaging studies in such cases often reveal hypodense or micronodular lesions, findings that can also be observed in metastatic liver disease or fungal infections. In this patient, cat scratch disease was clinically suspected, but the typical necrotizing stellate abscesses surrounded by palisading histiocytes were absent. A definitive diagnosis was achieved only through a combination of histopathological suspicion and microbiological confirmation of TB.

Similar cases in the literature underscore the diagnostic challenges posed by hepatic TB. Wyffels et al. reported cases where hepatic TB mimicked metastatic liver cancer or cholangiocarcinoma [7]. Likewise, Niyogi et al. described five cases of primary hepatic TB in which imaging studies initially suggested malignancy, with diagnoses ultimately confirmed through biopsy [8,9]. These examples reinforce the need for a high index of suspicion for TB in HIV-positive patients presenting with unexplained liver lesions.

Current guidelines recommend histopathological confirmation for extrapulmonary TB, as imaging alone is often insufficient [10]. Clinicians must remain vigilant for opportunistic infections in HIV-positive patients, which can further complicate diagnosis. Given the extent of involvement in this patient, disseminated TB was presumed, necessitating early initiation of therapy. Anti-TB treatment remains the cornerstone of management, typically involving an initial two-month regimen of isoniazid, rifampin, pyrazinamide, and ethambutol, followed by isoniazid and rifampin for an additional four months. This case underscores the critical importance of timely diagnosis and early initiation of treatment to prevent severe complications associated with disseminated TB in immunocompromised individuals.

## Conclusions

Extrapulmonary TB involving the liver and spleen should be considered in HIV-positive patients presenting with unexplained systemic symptoms such as persistent fever, weight loss, and fatigue. Because hepatic and splenic lesions may closely mimic malignancies or fungal infections, a definitive diagnosis requires combined histopathological and microbiological evaluation. Pathology plays a central role, as the identification of granulomatous inflammation guides targeted microbiological investigations, including PCR, for precise etiological confirmation.

Importantly, clinical improvement following corticosteroid therapy, often interpreted as IRIS, should not be relied upon in isolation without thoroughly excluding active infection. Early recognition and prompt initiation of anti-TB therapy are crucial to improve outcomes in immunocompromised patients, limiting disease progression and reducing the risk of severe complications.
